# TGA transcription factors—Structural characteristics as basis for functional variability

**DOI:** 10.3389/fpls.2022.935819

**Published:** 2022-07-26

**Authors:** Špela Tomaž, Kristina Gruden, Anna Coll

**Affiliations:** ^1^Department of Biotechnology and Systems Biology, National Institute of Biology, Ljubljana, Slovenia; ^2^Jožef Stefan International Postgraduate School, Ljubljana, Slovenia

**Keywords:** DOG1 domain, functional variability, intrinsically disordered regions, plant transcription regulation, post-translational modifications, structural characteristics, TGA transcription factors

## Abstract

TGA transcription factors are essential regulators of various cellular processes, their activity connected to different hormonal pathways, interacting proteins and regulatory elements. Belonging to the basic region leucine zipper (bZIP) family, TGAs operate by binding to their target DNA sequence as dimers through a conserved bZIP domain. Despite sharing the core DNA-binding sequence, the TGA paralogues exert somewhat different DNA-binding preferences. Sequence variability of their N- and C-terminal protein parts indicates their importance in defining TGA functional specificity through interactions with diverse proteins, affecting their DNA-binding properties. In this review, we provide a short and concise summary on plant TGA transcription factors from a structural point of view, including the relation of their structural characteristics to their functional roles in transcription regulation.

## Introduction

**FIGURE 1:**
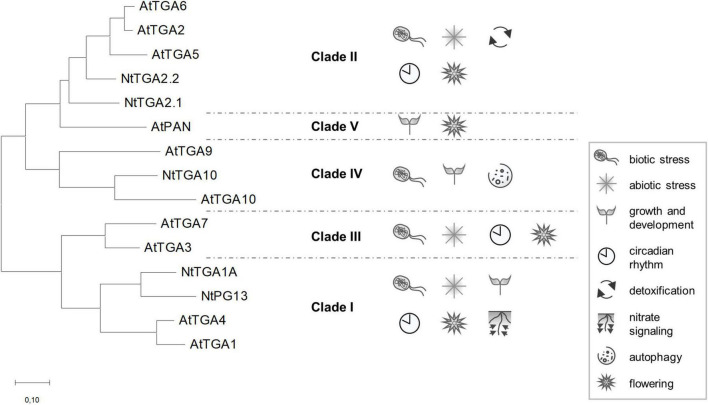
Unrooted phylogenetic tree of Arabidopsis and tobacco TGAs. Phylogenetic analysis of TGA factors shows an earlier separation of clades into two branches, one dividing into clades II, IV, and V, the other into clades I and III, indicating a closer evolutionary relationship between clade members in the same branch. TGA involvement in regulation of different processes, based on literature search, is represented for each clade. Sequence alignment by MUSCLE (Edgar, 2004) and phylogenetic analysis by the Maximum Likelihood method, based on the JTT matrix-based model (Jones et al., 1992), were conducted in MEGA7 (Kumar et al., 2016). The branch length scale represents the number of substitutions per site. Protein sequences with listed protein identification numbers were retrieved from UniProtKB (https://www.uniprot.org/): AtTGA1 (Q39237), AtTGA2 (P43273), AtTGA3 (Q39234), AtTGA4 (Q39162), AtTGA5 (Q39163), AtTGA6 (Q39140), AtTGA7 (Q93ZE2), AtPAN (Q9SX27), AtTGA9 (Q93XM6), AtTGA10 (E3VNM4), NtTGA1A (P14232), NtPG13 (Q05699), NtTGA2.1 (O24160), NtTGA2.2 (Q9SQK1), and NtTGA10 (Q52MZ2).

The *Arabidopsis thaliana* genome encodes for over 2,200 transcription factor genes, according to the Plant Transcription Factor Database^1^ yet few of them have been thoroughly characterized. The TGACG-binding (TGA) transcription factors were among the first plant transcription factors ever studied, their discovery dating back to the year of 1989 (Katagiri et al., 1989). Named after their hallmark binding site, the TGA factors became known for their regulation of defense-related genes through interaction with NON-EXPRESSOR OF PR-1 (NPR1) cofactor (Zhang et al., 1999), a salicylic acid receptor and master regulator of plant immunity (Wu et al., 2012; Backer et al., 2019; Wang W. et al., 2020). Among dicot plant species, the ten Arabidopsis TGA factors, AtTGA1-7, AtPERIANTHIA (AtPAN), and AtTGA9-10, have been investigated most thoroughly, next to five tobacco (*Nicotiana tabacum*) members, NtTGA1A, NtPG13, NtTGA2.1, NtTGA2.2, and NtTGA10. They are distributed into five clades (Jakoby et al., 2002), which are phylogenetically divided into two branches (Figure 1). Functional analysis of TGAs from different clades revealed not only their importance in biotic stress response (Zhang et al., 2003; Kesarwani et al., 2007; Zander et al., 2010; Sun et al., 2018), but also in regulation of gene expression connected to abiotic stress responses (Zhong et al., 2015; Fang et al., 2017; Herrera-Vásquez et al., 2021), developmental processes (Murmu et al., 2010; Maier et al., 2011; Wang et al., 2019), circadian rhythm (Zhou et al., 2015), detoxification (Fode et al., 2008; Mueller et al., 2008; Herrera-Vásquez et al., 2021), nitrate signaling (Alvarez et al., 2014; Canales et al., 2017), flowering (Thurow et al., 2005; Song et al., 2008; Maier et al., 2011; Xu et al., 2021), and autophagy (Wang P. et al., 2020) (Figure 1). Initially, the function of TGAs from clades I, II, and III was mainly associated with plant immunity, whereas the first reports on clade IV and V members revealed their role in regulating developmental processes (reviewed in Gatz, 2013). However, this apparent functional division is becoming less evident as an increasing number of reports show that most clades are involved in a variety of processes (Figure 1). For example, clade I TGAs have also been shown to be involved in regulating growth and development (Li et al., 2019; Wang et al., 2019), while clade IV TGAs are also important in biotic stress (Noshi et al., 2016; Venturuzzi et al., 2021).

A detailed review, integrating TGA transcription factor research, was published last in 2013 (Gatz, 2013). The complexity of TGA involvement in various molecular processes, the lack of research supporting the proposed mechanisms of action and limited reports on the relation between structure and *in vivo* function have become characteristic features of TGA studies and are the reason for our lack of knowledge about their mechanism of action. In order to understand how transcription factors operate, we must consider their structural characteristics as the underlying basis of protein activity. Here, we consider the importance of reported and *in silico* determined TGA structural features in defining their functional specificity and variability, focusing on the characterized TGA factors from Arabidopsis and tobacco.

## From TGA factor structure to their function

**FIGURE 2:**
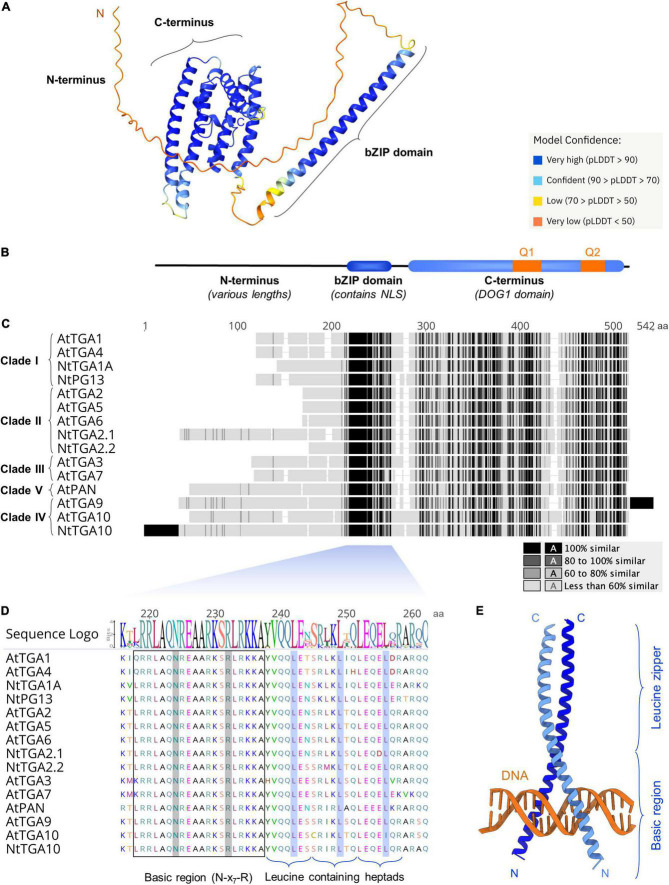
Among the three main TGA protein parts, the bZIP domain is the most highly conserved. **(A)** The AlphaFold generated 3D model of AtTGA1 (Jumper et al., 2021; Varadi et al., 2022) (pLDDT, AlphaFold per-residue confidence score) and **(B)** a schematic representation of TGA domain organization, showing the flexible N-terminus, the bZIP domain and the C-terminus, encompassing a putative Delay of Germination 1 (DOG1) domain. The nuclear localization signal (NLS) and glutamine rich regions Q1 and Q2 are indicated. **(C)** Multiple sequence alignment of ten Arabidopsis and five tobacco TGAs, with segments of high similarity or identity colored darkest and lowest similarity lightest, shows the bZIP domain retains the highest sequence identity throughout the whole protein sequence. In cases where sequence segments at N-terminal or C-terminal ends are not aligned to any of the other sequences, they are considered identical and are colored black. **(D)** A closer examination of the bZIP domain shows few variations in the basic region, while the three zipper heptads, with conserved leucine residue positions marked, show higher variability. The alignment and sequence logo were prepared and visualized with Geneious Prime 2020 (Kearse et al., 2012), using default parameters. **(E)** Structural model of the human FosB-JunD bZIP dimer in complex with DNA (5VPE entry in RCSB PDB) (Yin et al., 2017). The models in **(A,E)** were visualized in UCSF ChimeraX (Pettersen et al., 2021).

For more than 30 years after their discovery, the TGA protein three-dimensional (3D) structure remained a mystery and the first structural data have been published only recently in a breakthrough report providing a partial cryo-electron microscopy (cryo-EM) structure of AtTGA3 in complex with NPR1 (Kumar et al., 2022). Additionally, the novel artificial intelligence algorithm AlphaFold (AF) allows structure prediction without the availability of known similar structures (Jumper et al., 2021) and AF models for full-length Arabidopsis and tobacco TGAs are already available in the AF Protein Structure Database (Figure 2A; Varadi et al., 2022). In the following subchapters, we aim to connect literature reports with *in silico* analyses, to better understand the biological role of the three main structural parts of TGAs: The conserved basic region leucine zipper (bZIP) domain, the highly variable N-terminal part, from now on referred to as the N-terminus, and the C-terminal part (C-terminus), containing a putative Delay of Germination 1 (DOG1) domain (Figures 2A,B).

### The highly conserved DNA-binding domain

TGAs are members of the bZIP protein superfamily and represent a plant-specific subgroup, found in different species, including mosses and liverworts (Gutsche and Zachgo, 2016; Gutsche et al., 2017). bZIP proteins are defined by their DNA-binding and dimerization region known as the bZIP domain, which is highly conserved among plants and even across kingdoms (Jindrich and Degnan, 2016). Multiple sequence alignment of Arabidopsis and tobacco TGAs shows the bZIP domain as the region of highest protein sequence identity, regardless of plant species (Figure 2C). The bZIP domain determines DNA-binding specificity and serves as a nuclear localization signal (Figure 2B; van der Krol and Chua, 1991; Deppmann et al., 2004). It consists of two regions, the basic region and the leucine zipper. Hydrogen bond formation with the major DNA groove is facilitated through the basic region, which contains an invariant N-x_7_-R/K motif (Dröge-Laser et al., 2018). Nineteen out of 20 amino acids of the TGA basic region, including the N-x_7_-R motif, remain identical in all aligned Arabidopsis and tobacco sequences, with a few clade/species-specific differences present only in the first residue (Figure 2D).

bZIP proteins bind target DNA as dimers, with combinatorial homo- or heterodimerization at the DNA-binding site granting them broad variability in regulation of physiological responses (Deppmann et al., 2006; Rodríguez-Martínez et al., 2017). The leucine zipper confers dimer formation and determines dimerization specificity. It consists of repetitive seven-amino acid units, called heptads. Each heptad contains a conserved leucine residue at its fifth position (Landschulz et al., 1988; Deppmann et al., 2006). TGA bZIP domains have three leucine zipper heptads, which show higher variability than the basic region, yet retain the conserved leucines. The only exception is AtTGA10, where the third leucine is replaced with isoleucine (Figure 2D). The number of heptads is among the lowest compared to other Arabidopsis bZIP proteins (Deppmann et al., 2004), rendering the 41 aa bZIP domains in TGAs considerably shorter from the typical 60–80 aa bZIP length (Jindrich and Degnan, 2016). Additionally, the leucine zippers of TGAs contain destabilizing residues at dimer contact sites, making the zipper formation less stable (Deppmann et al., 2004). The extent to which dimerization stability affects transcription factor binding time at its specific motif is not known and unstable interactions might shorten DNA-binding times, resulting in a lower number of generated transcripts (Swift and Coruzzi, 2017).

During DNA-binding the bZIP domains of two proteins grip the DNA segment in a scissor-like fashion, while attaining an alpha-helical fold (Vinson et al., 1989; Ellenberger et al., 1992), as shown in the human FosB-JunD-DNA complex 3D structure (Figure 2E; Yin et al., 2017). The role of the leucine zipper in TGA dimerization had been demonstrated by switching the leucine zipper in NtTGA2.2 for a zipper from the human Jun bZIP protein, which prevented heterodimerization with NtTGA2.1 (Thurow et al., 2005). However, deletion of 93 aa from AtTGA2 N-terminus, including the entire bZIP domain, still allowed homodimer formation (Boyle et al., 2009) and multiple reports have shown that TGA dimerization depends significantly on other protein parts as well. A dimer stabilization region had been identified in the C-terminus of tobacco NtTGA1A, located between 178 and 373 aa (Katagiri et al., 1992). Formation of stable contacts through the TGA C-terminus was recently confirmed with cryo-EM, which revealed homodimerization of AtTGA3 C-termini in the AtTGA3-NPR1 complex structure (Kumar et al., 2022). Moreover, deleting the residues from 146 to 330, spanning more than half of the protein C-terminus, abolished the DNA-binding activity of AtTGA2 (Johnson et al., 2008), which could be due to hindered dimerization. Additionally, protein interaction analyses *in vitro* and *in vivo* showed that TGAs can form homodimers, heterodimers as well as higher order complexes (Niggeweg et al., 2000; Schiermeyer et al., 2003; Boyle et al., 2009) and the oligomerization properties of AtTGA2 seem to be dependent on the region spanning its N-terminus and bZIP domain (Boyle et al., 2009).

Recognition of only a short DNA sequence is usually sufficient for transcription factor binding (Kribelbauer et al., 2019). The TGACG pentamer is the common TGA dimer binding site and sufficient for their binding (Katagiri et al., 1992; Schindler et al., 1992; Izawa et al., 1993). ChIP-seq analysis of AtTGA2 revealed that 55% of significantly enriched regions in the Arabidopsis genome contained the TGACGTCA palindrome, while all carried at least the TGACG core motif (Thibaud-Nissen et al., 2006). The palindrome was also determined as the representative binding motif of AtTGAs in DAP-seq data (O’Malley et al., 2016). In addition, tandem TGACG repeats, such as the *activating sequence-1* (*as-1*) or *as-1*-like elements, allow more options regarding the binding stoichiometry. For example AtTGA2, AtTGA5, NtTGA2.1, NtTGA2.2, and NtTGA10, can bind tandem repeats in two-dimer complexes (tetramers). Although past reports indicated that AtTGA1, AtTGA3, and NtTGA1A prefer single-dimer formation (Lam and Lam, 1995; Niggeweg et al., 2000; Schiermeyer et al., 2003), Kumar et al. (2022) show single and double occupancy of tandem repeats in the *Pathogenesis related-1 (PR-1)* promoter by AtTGA3 in electrophoretic mobility shift assays. The single-occupancy band is depleted in the presence of NPR1, which supershifts the double-occupancy band. The AtTGA3-NPR1-DNA complex structure consists of four AtTGA3 and two NPR1 proteins, where an NPR1 dimer connects two DNA-bound AtTGA3 dimers (Kumar et al., 2022). The spacing between tandem repeats is also important, as it affects element recognition, binding affinity and TGA transcription activation ability (Krawczyk et al., 2002). Moreover, TGA paralogues have been shown to occupy the A-box (TACGTA), C-box (GACGTC), G-box (CACGTG), and T-box (AACGTT) motifs with different affinities (Izawa et al., 1993; Wang et al., 2019).

Heterodimerization of transcription factors with different binding preferences can, undoubtedly, result in distinct DNA-binding specificities and affinities (Rodríguez-Martínez et al., 2017). NtTGA2.1/NtTGA2.2-NtTGA1A heterodimers have been recruited to a single TGACG motif (Niggeweg et al., 2000). Homodimer binding can be stabilized by the presence of other TGA homodimers at the tandem occupancy site (Lam and Lam, 1995). Besides the motif sequence, adjacent sequence regions are also important for binding, as they determine intrinsic DNA properties and consequently the transcription factor binding affinity (Kribelbauer et al., 2019). For example, when compared to the *as-1*-like element, the affinity of AtPAN was stronger for the 33 bp long AAGAAT motif, characterized by an AAGAAT sequence upstream of a single TGACG pentamer (Gutsche and Zachgo, 2016). Additionally, local adjustments of TGA concentration, for example through specific subnuclear localization, may contribute to successful binding to suboptimal binding sites (Kribelbauer et al., 2019).

### Staying flexible through N-terminus

The N-terminus is likely a major contributor in defining TGA functional specificity. It is the least conserved part of the TGA structure, with a high variability in amino acid sequence and length (Figure 2C). Studies analyzing the TGA N-terminus function indicate its various roles, but only a few of them have been corroborated by further analysis. Due to its relatively high acidic amino acid content (9–24%, calculated as the percentage of aspartic and glutamic acid residues), it was proposed that it likely takes on a transcription regulation function (Katagiri et al., 1989). Acidic regions are common to many transcription activation domains and, according to the model proposed by Staller et al. (2018), help exposing the hydrophobic residues to facilitate contact formation with coactivators. In support of this, removing the N-terminus of NtTGA1A diminished its transcription activation ability in tobacco cotyledons (Neuhaus et al., 1994).

**FIGURE 3:**
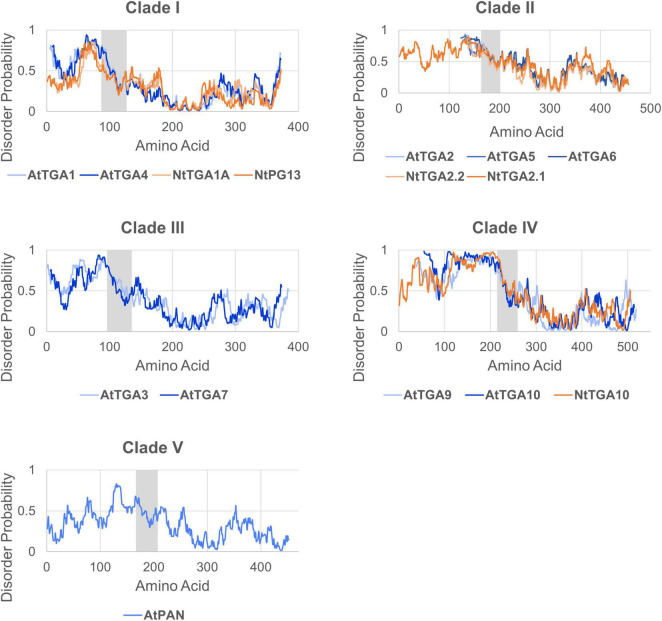
The N-termini of TGAs are intrinsically disordered. Representation of intrinsic disorder regions of full-length TGA amino acid sequences from Arabidopsis and tobacco, created based on IUPred2 prediction algorithm results (Mészáros et al., 2018). The N-termini of analyzed TGAs show generally high (>0.5), yet clade-specific pattern of intrinsic disorder probability, while the disorder probability is considerably lower in their C-termini. Charts representing TGAs from the same clade were aligned based on the conserved bZIP domain, which is shown as grey area.

Because activation domains often interact with a variety of structurally distinct coactivators, they are usually intrinsically disordered as well, thus their 3D structure is hard to determine (Staller et al., 2018). In line with this, the TGA N-terminal region is mostly unstructured in the AF models (Figure 2A) and has an overall high probability of disorder (>0.5) with variably interspaced more structured sections according to three intrinsic disorder predictors, IUPred2 (Figure 3; Mészáros et al., 2018), PrDOS (Ishida and Kinoshita, 2007) and SPOT-disorder (Hanson et al., 2017) (Supplementary Figure 1). The intrinsic disorder pattern as well as the N-terminus length seem largely clade-dependent. Clade I and III TGAs all harbor medium length N-termini of about 75–100 aa, which, according to two of the prediction algorithms, share a higher probability of disorder near the basic region, with disorder decreasing further away from the bZIP domain. Most clade II members exhibit short N-termini of about 40–50 aa, with a high disorder-probability spanning their whole length. Clade IV and clade V member N-termini are the longest, reaching 216 aa in NtTGA10. While intrinsic disorder is higher in clade IV TGA N-termini, the AtPAN N-terminus seems more structured based on IUPred2 and PrDOS predictions (Figure 3 and Supplementary Figure 1).

Electrophoretic mobility shift assay results have shown that the long N-terminus of NtTGA2.1 enabled weak binding to the *as-1* element, while the binding of shorter NtTGA2.2 was stronger. Furthermore, shortening the NtTGA2.1 N-terminus increased the protein DNA-binding stability (Niggeweg et al., 2000). Modulation of protein-DNA interaction stability therefore may be one of the N-terminus features. Additional stabilizing elements in DNA-binding motif vicinity can stabilize the protein-DNA interactions through parts other than the DNA-binding domain. Motif recognition and binding stabilization through the N-terminus have been shown in other transcription factors. For instance, the N-terminal arm of the *Drosophila melanogaster* Hox homeodomain factor stabilizes the binding to DNA by inserting the positively charged amino acids within the arm into the minor DNA groove (Abe et al., 2015).

While several discrepancies regarding the mechanisms of AtTGA-mediated transcriptional regulation in cooperation with NPR1 remain (discussed previously in Gatz, 2013), the study from Boyle et al. (2009) indicated that the TGA N-terminus is important for determining its activation/repression function. In accordance with the results from Zhang et al. (2003) and Kesarwani et al. (2007), who show that AtTGA2 acts as a constitutive repressor, modulating basal promoter activity of the *PR-1* gene, the AtTGA2 N-terminus assumes a non-autonomous repression function and proved important also for AtTGA2 oligomerization at the DNA-binding site (Boyle et al., 2009). However, AtTGA2 interaction with NPR1 in the presence of salicylic acid activates gene expression (Rochon et al., 2006). In the proposed NPR1-AtTGA2 activation complex, NPR1 prevents the DNA-binding of AtTGA2 oligomer and negates the AtTGA2 repression function through interaction with its N-terminus (Boyle et al., 2009).

Interestingly, Gutsche and Zachgo (2016) described the role of AtPAN N-terminus in connection to its redox-sensitive DNA-binding. Its unique feature is the presence of five cysteine residues, dispersed throughout the N-terminus. AtPAN binds the AAGAAT motif in reducing conditions, while an oxidizing environment diminishes this interaction. Mutations of all AtPAN cysteines, including Cys340 in the C-terminus or the complete removal of N-terminus, both prevented such redox-dependent motif-binding (Gutsche and Zachgo, 2016). The activity of the N-terminus was further confirmed *in planta*. The expression of either AtPAN N-terminus deletion mutant, AtPAN with mutated N-terminal Cys68 and Cys87, or of AtPAN with substituted of all N-terminal cysteines to serines could not complement the *pan* knockout plant phenotype (Gutsche and Zachgo, 2016). Nevertheless, the mechanisms of redox-dependent sensitivity based on N-terminal cysteines remain to be elucidated.

On the other hand, the TGA N-terminus effects on interactions with other proteins should also be considered. The AtTGA2 N-terminus can be bound by the copper chaperone induced by pathogens (CPP) and the AtTGA2-CPP interaction enhances AtTGA2 binding to the *PR-1* promoter (Chai et al., 2020). The N-terminal half of AtTGA3, including the bZIP domain, interacts with the WRKY53 transcription factor (Sarkar et al., 2018), while it also enhances AtTGA3 interaction with NPR1 in yeast (Zhou et al., 2000). Yeast two-hybrid assays of AtPAN and AtTGA3 deletion mutants indicated that their N-termini strengthen interactions with ROXY1 CC-type glutaredoxin (Li et al., 2011), however, these results have not been confirmed with quantitative analyses.

### C-terminus: Glutamine rich regions and the DOG1 domain

TGAs share a relatively conserved C-terminus of about 250 aa in length. Overall, it has a lower intrinsic disorder-probability in all clades (Figure 3 and Supplementary Figure 1) and contains two 20–30 aa long regions rich in glutamine residues, designated Q1 and Q2 (Figure 2B; Katagiri et al., 1989; Gatz, 2013). Glutamine-rich regions occur in transcription activation domains and can modulate transcription activation through unknown mechanisms (Arnold et al., 2018). Activation and/or repression function of individual TGAs is therefore likely the result of both N- and C-terminal contributions of the same protein. As described above, the TGA N-terminus is important for modulation of protein-protein interactions. However, the C-terminus has been identified as the main protein-protein interaction region in several studies. It is sufficient for interaction of AtTGA2 and AtTGA3 with NPR1 (Fan and Dong, 2002; Johnson et al., 2008; Kumar et al., 2022). AtPAN and AtTGA3 interact with ROXY1 primarily through the Q2 and the intervening region, which represents the first third of their C-termini (Li et al., 2011).

Consistent with the TGA AF model (Figure 2A), the cryo-EM and crystallographic data presented by Kumar et al. (2022) show that the 3D structure of AtTGA3 C-terminus (aa 166–377) is predominantly alpha-helical, containing five longer and three shorter alpha-helices that are connected with flexible linkers. The alpha-helices envelop a single molecule of palmitic acid, the role of which has yet to be elucidated. The C-terminus is involved in AtTGA3 dimerization as well as interaction with the ankyrin repeat region of NPR1, leading the authors to refer to it as NPR1-interacting domain (NID). The NID forms contacts with NPR1 through four residues near the center of the AtTGA3 C-terminus sequence (Glu263, Pro264, Thr266, and Asp267) and four residues close to its C-terminal end (Thr351, Thr352, Arg353, and Arg357). Additionally, by using a series of chimeric AtTGA1/AtTGA2 proteins, Després et al. (2003) show the importance of AtTGA2 C-terminal aa 236–266 in establishing the interaction with NPR1. Furthermore, the NPR1 Broad-Complex, Tramtrack, and Bric-a-brac/Pox virus and Zinc finger domain was shown to interact with AtTGA2 N-terminus (Boyle et al., 2009). While the cryo-EM structures of AtTGA3 bZIP and N-terminus could not be determined likely due to flexible linker connecting them to the NID (Kumar et al., 2022), it would be interesting to compare their involvement in the TGA-NPR complex.

The TGA C-terminus contains also the DOG1 domain, which spans most of the region according to the ExPASy Prosite domain prediction tool (Sigrist et al., 2013). The domain name originates from the Arabidopsis DOG1 protein, a plant-specific protein involved in seed dormancy control (Bentsink et al., 2006). DOG1 was also identified as a microprotein, a transcription factor-like protein of low molecular weight without the DNA-binding ability that could be involved in modulation of TGA activity (Magnani et al., 2014). Circular dichroism spectra of a recombinant DOG1 revealed it to be an alpha-helical protein as well, containing a heme-binding site important for DOG1 function (Nishimura et al., 2018). Despite low sequence identity between AtTGAs and DOG1, some DOG1 domain residues remain conserved and may contribute to the final protein fold or any key structural and functional characteristics (Sall et al., 2019), indicating the possibility of heme-binding activity also in TGAs. Phylogenetic analyses have shown that TGAs form a monophyletic group outside of DOG1 family members, which include DOG1 and five DOG1-like (DOGL) proteins (Nishiyama et al., 2021). The presence of conserved amino acid residues related to Calmodulin (CaM) binding in the C-terminus in both protein groups, indicates that DOG1 could act as a CaM-binding domain in AtTGA transcription factors (Sall et al., 2019). CaM is an important calcium (Ca^2+^) sensor and affects a number of cellular processes in response to increased concentrations of free Ca^2+^ (Bergey et al., 2014). Several TGAs have been identified as CaM interactors (Popescu et al., 2007). The CaM/Ca^2+^ complex enhanced AtTGA3 binding to TGACG elements *in vivo* and *in vitro* by direct protein-protein interaction (Szymanski et al., 1996; Fang et al., 2017), signifying a close connection of TGA transcription regulation with Ca^2+^ influx, a primary occurrence following stress-related events in plant cells (Tian et al., 2020).

Additionally, interactions with a variety of structurally distinct proteins have been shown to affect TGA activity, but the protein part important for the interaction has not yet been identified. For instance, clade II AtTGAs interact with WRKY50 transcription factor to cooperatively activate *PR-1* gene expression (Hussain et al., 2018), or with NPR1 paralogues NPR3 and NPR4 to repress the expression of *SAR DEFICIENT 1* (*SARD1)* and *WRKY70* (Ding et al., 2018). Recently it has been shown that AtTGA5 and AtTGA7 interact with CYCLIN-DEPENDENT KINASE 8 (CDK8), involved in the recruitment of RNA polymerase II (Chen et al., 2019), and AtTGA2 with HIGH OSMOTIC STRESS GENE EXPRESSION 15 (HOS15) corepressor (Shen et al., 2020). All Arabidopsis TGAs interact with ROXY glutaredoxins, albeit with different binding affinities (Li et al., 2009; Murmu et al., 2010; Zander et al., 2012). Being able to interact with various cofactors suggests a certain flexibility in the binding region itself or the presence of multiple binding sites. Considering the C-terminus to be fairly structured, it could contain more than one protein-binding region. To further facilitate interaction specificity and modulate binding affinity of different TGA-interactor combinations, additional contact sites are likely mediated by the variable N-terminus.

### What is the role of post-translational modifications?

Post-translational modifications (PTMs) are known to be important in regulation of protein function and can affect protein activity on many levels, including dimerization, DNA-binding or protein interactions. TGAs have been found to be subjected to phosphorylation (Kang and Klessig, 2005; Kim et al., 2022), S-nitrosylation, S-glutathionylation (Lindermayr et al., 2010; Gutsche and Zachgo, 2016) and most notably redox-dependent regulation through disulphide bond formation (Després et al., 2003; Lindermayr et al., 2010; Gutsche and Zachgo, 2016). Phosphorylation was among the first PTMs studied in TGAs. Clade II AtTGAs and to a lesser extent AtTGA3, but not clade I AtTGAs, can be phosphorylated by casein kinase II (CK2) and experiments with AtTGA2 deletion mutants revealed the phosphorylation site to be within the first 20 aa of its N-terminus. The CK2-mediated phosphorylation of AtTGA2 reduced its DNA-binding activity (Kang and Klessig, 2005). On the other hand, the clade I AtTGAs have been shown to be phosphorylated by BR-INSENSITIVE2 (BIN2) at their C-terminus. This phosphorylation destabilized AtTGA4 and inhibited its interaction with NPR1 *in vivo* (Kim et al., 2022).

TGAs seem tightly connected to redox-dependent regulation and several studies focused on examining the importance of TGA cysteine residues. Clade II AtTGAs contain only one cysteine in their C-terminus, but its function remains unknown (Huang et al., 2016; Findling et al., 2018). AtPAN retains five cysteines in the N-terminus alone, which are involved in redox-dependent modulation of AtPAN DNA-binding, while the S-glutathionylation of the Cys340, localized in a putative transcription activation domain in AtPAN C-terminus, indicates that additional mechanisms could modify its activity post-translationally (Gutsche and Zachgo, 2016). Clade I AtTGAs contain four cysteines, two of which are unique and were found to facilitate redox-dependent interaction with NPR1 in the presence of salicylic acid (Després et al., 2003), with the redox regulation proposedly mediated by nitric oxide (Lindermayr et al., 2010). Furthermore, substitutions of the same cysteines prevent clade I AtTGA interactions with the NPR-family Blade-on-Petiole (BOP) proteins (Wang et al., 2019). However, Budimir et al. (2021) showed that the reduction of cysteines in AtTGA1 may not affect its function in salicylic acid-dependent gene expression. The conflicting results regarding clade I redox regulation were discussed recently (Li and Loake, 2020), and although a tight connection between TGAs and the intracellular redox state is clearly important for their activity, the role of TGA cysteine residues is still a subject of debate.

## Discussion

**FIGURE 4:**
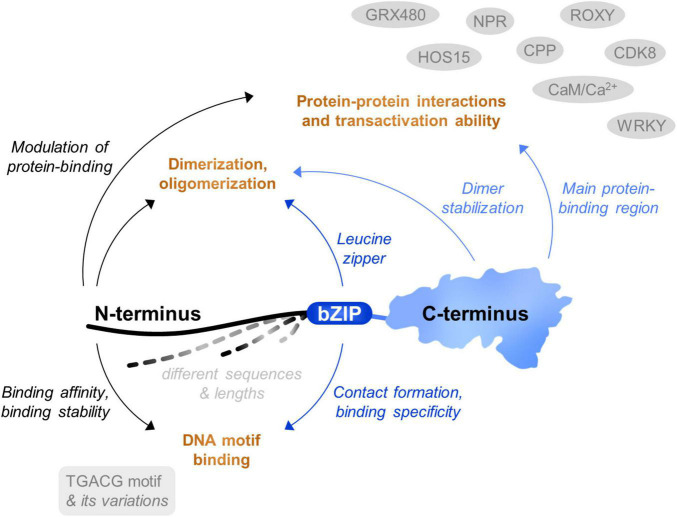
Schematic representation of TGA protein parts contribution to TGA function. All three protein parts of TGAs are multifunctional, each involved in several tasks connected to their interaction with target DNA motifs, dimerization and/or oligomerization and protein-protein interactions with transcription factors, cofactors or other proteins, resulting in a specific shift in gene expression activity.

Although TGAs represent a relatively small group of regulatory proteins, they are able to affect a wide range of cellular processes. The three main TGA protein parts have individual yet overlapping roles, jointly contributing to the functional variability of each paralogue (Figure 4). Thus far, the link between TGA structural characteristics and plant phenotype is not well studied. Extensive work has been dedicated to understanding how TGAs bind to DNA, as well as to which genes they regulate (Gatz, 2013). However, while one area of studies focuses on the biochemistry of binding, the other is concentrated mainly on the function of TGAs in interaction with other proteins, using knockout plants. The use of high-throughput DNA-binding methodologies in TGA studies can be challenging due to their low abundance in plant tissue and the question of how different TGAs with similar DNA-binding preferences can regulate so many different functions remains to be elucidated.

Specific studies should be designed to understand how the ability to form homo/heterodimers, tetramers or higher order complexes with other proteins at promoter regions affects target transcription and the physiological response of the plant. Most of the available molecular information is based on the studies of the *PR-1* promoter, which may not be representative. Additional gene models should be developed to study the TGA mechanism of action *in vivo*. Analyzing local DNA structural features *in silico* (Li et al., 2017; Samee et al., 2019) should also be considered for identification and analysis of TGA binding specificities. Suboptimal binding sites can contribute to TGA function as well, depending on local transcription factor molecule concentration, determined by their spatio-temporal gene expression and non-redundant subnuclear distribution (Kribelbauer et al., 2019). The mechanisms regulating TGA abundance, however, are not well understood. TGAs are differentially regulated at the expression level subsequent to pathogen infection, abiotic stress and show tissue specific expression (Chen et al., 2018; Wang et al., 2019; Seo et al., 2020). In addition they are subjected to complex post-transcriptional (Pontier et al., 2002) and post-translational processes (Kang and Klessig, 2005; Lindermayr et al., 2010; Gutsche and Zachgo, 2016).

In order to thoroughly understand the role and interplay of different TGA clade members, it is imperative to recognize key structural differences between them, individually and in higher order complexes. Resolving the complete TGA factor 3D structure in complex with their various protein interactors at target promoters would provide the basis for further experiments in studying TGA activity. Development of cryo-EM methods, which are already reaching atomic resolution (Yip et al., 2020), proved valuable in structural analysis of complexes and will continue playing an important role in the future of structure determination. Alternatively, computational modeling algorithms, such as AF (Jumper et al., 2021), can provide a useful solution to understand the structure-function relationship better, when obtaining structures experimentally proves difficult (Greener et al., 2019; Aderinwale et al., 2020).

## Author contributions

ŠT, KG, and AC conceptualized the idea. ŠT performed *in silico* analyses and wrote the initial manuscript draft. All authors contributed to the writing and revision of the manuscript.

## Footnotes

1
http://planttfdb.gao-lab.org
