# Supplementary material for: TGA transcription factors—Structural characteristics as basis for functional variability
Source: Front Plant Sci. 2022 Jul 26;13:935819. doi: 10.3389/fpls.2022.935819 (PMC9360754; doi:10.3389/fpls.2022.935819)
Supplement: Supplementary file 1 [file Image_1.PDF]

Supplementary Material

1 Supplementary Figures

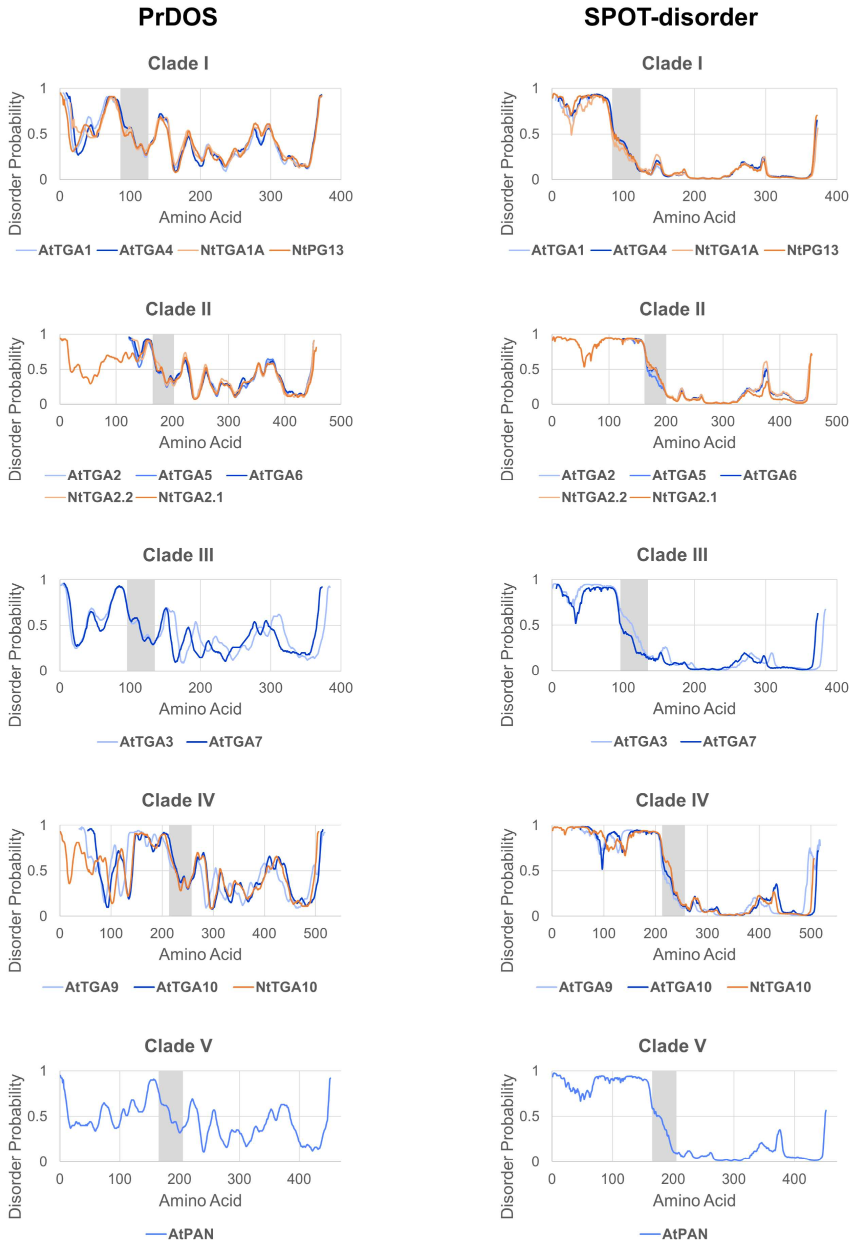

Supplementary Fig. 1. Intrinsic disorder in TGAs according to PrDOS and SPOT-disorder algorithms. Additional representation of intrinsic disorder regions of full-length TGA amino acid sequences from Arabidopsis and tobacco, created based on PrDOS (left panel) and SPOT-disorder (right panel) prediction algorithm results (Ishida and Kinoshita, 2007; Hanson et al., 2017). Charts representing TGAs from the same clade were aligned based on the conserved bZIP domain, which is shown as grey area.
